# Exogenous RNA as a Regulatory Signal during a Plant’s Interaction with the Biotic Environment: An Evolutionary Perspective and Future Applications in Agriculture

**DOI:** 10.3390/plants10030532

**Published:** 2021-03-12

**Authors:** Sergey Ivashuta, Alberto Iandolino, Greg Watson

**Affiliations:** Bayer AG, Chesterfield, MO 63017, USA; alberto.iandolino@bayer.com (A.I.); greg.watson@bayer.com (G.W.)

**Keywords:** exogeneous RNA, environmental RNAi, plants, evolution

## Abstract

Environmental RNAi (eRNAi) is a sequence-specific regulation of endogenous gene expression in a responsive organism by exogenous RNA. While exogenous RNA transfer between organisms of different kingdoms of life have been unambiguously identified in nature, our understanding of the biological significance of this phenomenon remains obscure, particularly within an evolutionary context. During the last decade multiple reports utilizing various mechanisms of natural eRNAi phenomena have been attempted to develop new agricultural traits and products including weed, disease and insect control. Although these attempts yielded mixed results, this concept remains extremely attractive for many agricultural applications. To better utilize eRNAi for practical applications, we would like to emphasize the necessity of understanding the biological significance of this phenomenon within an evolutionary context and learn from nature by developing advanced tools to identify and study new cases of exogeneous RNA transfer and eRNAi. In this opinion article we would like to look at the exogeneous RNA transfer from an evolutionary perspective, propose that new cases of exogeneous RNA transfer still remain to be identified in nature, and address a knowledge gap in understanding the biological function and significance of RNA transfer. We believe such approach may eventually result in a more successful use of this phenomenon for practical applications in agriculture.

Regulatory RNAs (RegRNA) form a diverse class of non-coding RNA molecules in eukaryotic and prokaryotic organisms that regulate gene expression through a sequence-specific interaction with RNA or DNA. The significance of many classes of regRNAs in regulating the expression of complex networks of genes in organisms during development or in response to stresses has been described in numerous publications and reviewed elsewhere [1]. The most well-known classes of regRNAs found in most eukaryotic organisms include small RNA (sRNA) (miRNA, siRNA, and other subclasses, reviewed elsewhere) and long non-coding RNAs (lncRNA) that regulate gene expression on transcriptional, post-transcriptional and translational levels [2,3]. In prokaryotes, an expanding list of regRNAs include multiple cis- and trans-encoded sRNAs involved in regulation of gene expression by complementary base pairing with target RNAs or sequestration of regulatory proteins, and more recently discovered CRISPR RNAs (crRNAs) that play a role in defense against foreign DNA [4,5,6]. It should be noted that the prokaryote and eukaryotic regRNA systems follow separate rules and evolutionary paths and are not functionally interchangeable. Overall, the regulatory role of RNAs in organisms of all kingdoms of life is abundant and their functions are diverse. 

In addition to regulation of endogenous gene networks within an organism, another phenomenon that draws the attention of many biologists can be described as the regulation of gene expression in a receptive organism by exogenous RNA. Such sequence-specific regulation in a responsive organism by exogenous RNA is usually referred to as inter-kingdom or cross-kingdom RNA and/or environmental RNAi (eRNAi). The eRNAi term that in our opinion reflects a broader scope of this phenomenon and will be used in this publication to describe any cases of exogenous regRNA transfer in nature or designed and engineered in a laboratory. The first reported case of eRNAi was a regulation of gene expression by ingestion of exogenous dsRNA or bacterially-expressed dsRNA by *C. elegans* [7]. It was also shown that a non-coding RNA naturally expressed in *E. coli* is taken up by *C. elegans* and loaded into the RNAi machinery and down-regulates the *che-2* gene thus impairing the nematodes’ ability to find food [8]. 

A growing number of reported examples of eRNAi describing RNA transfer between plants and microorganisms, plants of different species, and between plants and herbivorous insects can be found in several reviews [9,10]. The evolutionary aspect of establishing RNA-based signaling between different organisms as well as the possibility of utilizing the eRNAi knowledge for practical applications make this phenomenon intriguing and worthy of detailed investigation. Recently reported cases of natural eRNAi, as well as examples of engineered eRNAi suggest that exogenous cross-kingdom RNA regulation may be widespread in nature; however, the overall picture of the eRNAi role in living organisms remains fragmented and the significance of this phenomenon in nature on a global scale is yet to be fully understood. 

Plants occupy a unique place in the Earth’s ecosystem by continuously interacting with numerous pathogenic or symbiotic microbes and herbivorous invertebrates, and thus forming the base of the food chain pyramid. This constant interaction creates numerous opportunities for an RNA transfer mechanism as a communication or regulatory signal between plants and biotic environment to evolve. Regulation of gene expression by exogenous RNA delivered to plant cells by gray mold during this plant–microbe interaction is one of several compelling examples of eRNAi [11]. Plants transporting sRNAs into microbes was identified in another plant-fungal pathogen interaction. In this example, where *Botrytis cinerea* delivered small RNAs into plant cells silencing host immunity genes, while the host plant delivered sRNAs to *Botrytis* to target fungal genes and attenuate fungal pathogenicity [12]. A practical technological application of this type of regulatory RNA transfer was demonstrated when lettuce plants were engineered to produce siRNAs targeting vital genes of the oomycete pathogen *Bremia lactuca* resulting in significant reduction in fungal growth and sporulation [13]. However, in spite of the growing number of reported cases of exogenous RNA signaling between plants and fungi, RNA exchange between plant and fungal pathogen is not always detected [14]. The lack of consistent demonstration/detection of transferred RNA raises questions as to the level of conservation existing for exogenous RNA-base signaling mechanisms in plant–fungal interactions. 

While the bidirectional eRNAi between plants and fungi relies on intimate cellular contact [15], the active exchange of regRNAs and other effectors via highly specialized extracellular vesicles and an extensive similarity in silencing machinery [9,16], the transfer of regRNA between plants and insects likely relies on different mechanisms. In plant–insect interactions, strong eRNAi responses have been found in some insect orders such a Coleoptera, but many lepidopteran species seem to be totally or partially refractory to eRNAi [17] suggesting different evolutionary paths to evolve responsiveness to exogenous RNA. Ingestion of corn tissue engineered to express long double-stranded RNAs (dsRNAs) with a sequence complimentary to essential insect genes in corn rootworm (CRW, *Diabrotica virgifera virgifera* LeConte) resulted in systemic down regulation of target genes and insect death [18]. This was the first clear demonstration of eRNAi between plants and insects. Interestingly, CRW is capable of dietary uptake of not only transgenically-expressed dsRNA but also numerous naturally-produced plant long dsRNA (40 bp and longer) but not small RNAs [19]. Plant-derived long dsRNAs were processed into functional insect siRNAs by the insect RNAi machinery and could account for a significant portion (up to 12%) of the overall siRNA pool in CRW cells [19]. At the same time, lepidopteran insects fed under the same conditions did not have any detectable plant-derived siRNAs suggesting a lack of plant dsRNA uptake [19]. Even within the same insect species, non-responsiveness to eRNAi has been observed. For example, a CRW population that was non-responsive to eRNAi was generated that lacked the ability to uptake long dsRNA from plant-based food sources [20]. This insect line had no obvious detrimental developmental effects under laboratory conditions, although we cannot exclude that eRNAi ability may have played a role in the organism in a more complex natural environment. While engineered eRNAi in the form of topically applied or transgenically expressed regRNA with a sequence complementary to insect target genes can help protect plants from insect pests, it is not clear what role eRNAi plays in plant defense against insect pests in nature. Thus, plants can be both donors and recipients of exogenous regRNA, actively or passively transferring regRNA into interacting organisms.

Inspired by examples of eRNAi found in nature and several cases of engineered eRNAi, there is increasing interest in leveraging the eRNAi for a broader range of practical applications in agriculture utilizing either plant incorporated or topical approaches. Plants have endogenous mechanisms to amplify regRNA signals and, in some cases, spread it systemically to distant cells or tissues [4] and this attribute may enhance the potential to exploit engineered eRNAi for crop protection applications. Exogenous regRNA-based strategies conceived to control herbicide resistant weeds [21] relied not only on the efficient uptake of topically applied regRNA but also on the systemic movement of the silencing effectors. In spite of recent advances in our understanding of factors limiting the practical application of topical eRNAi for weed control, technical solutions still need to be identified to overcome significant physicochemical and molecular barriers limiting the widespread activity of exogenously applied regRNAs [22]. For example, current technologies limit efficient delivery of siRNAs to cells in developing or mature leaves, leaving meristematic regions out of reach [23,24]. Additional cases of exogenous regRNA application to leaf surface of model plants have been reported and demonstrated the suppression of transgene or endogenous genes [25,26,27]. Systemic gene silencing initiated from localized topical delivery of sRNAs has been reported in *Nicotiana benthamiana* plants overexpressing GFP, a process that seems to be dependent, at least in part, on the size and abundance of the siRNA effector as well as on the relatively high expression of the target gene [22,23,28,29]. Our current understanding of the mechanisms restricting systemic RNAi-mediated silencing of endogenous genes after topical dsRNA application is limited. 

In order for exogenous regRNA to achieve a noticeable regulatory effect in heterologous organisms, the regRNA not only needs to be efficiently taken up by recipient cells in a biologically relevant concentration in the host, but also must be protected from intracellular degradation and be compatible with host RNA regulatory biogenesis pathways and silencing machinery. Therefore, in addition to exogenous regRNA bioavailability, the differences in compatibility with host machinery, including efficient loading into the host silencing complex along with systemic spread, can also be factors contributing to the variability in response to eRNAi. Several published examples of bidirectional plant fungal eRNAi demonstrated the importance of exosome-based active delivery of fungal sRNAs [15]. Learning from these naturally-occurring examples of eRNAi can help to develop a strategy for effective engineered eRNAi for specific practical applications. 

In nature, plants co-exist with a diverse set of microbes such as archaea, bacteria, fungi, protists and viruses forming a complex consortia called the “holobiont”. In such consortia where holobiont members cohabitate in close proximity for millions of years, co-evolution likely results in developing mutualistic, commensalistic or pathogenic interactions [30,31]. Unlike plant-fungus interactions where exogenous regRNA exchange and eRNAi have been clearly demonstrated, to our knowledge there is no reported cases of eRNAi between plants and prokaryotic microorganisms. However, as found with fungal pathogens, prokaryote pathogen-plant interactions have been shown to involve effectors (e.g., type-III secretion system effectors) [32]; and some of these effectors have the ability to bind RNA. Many bacteria are also able to form extracellular vesicles that can carry regRNA that could be a universal mechanism for RNA signaling in the holobiont and it has been proposed that sRNA may play a role in eRNAi between prokaryotic microbes and eukaryotic holobiont hosts [33,34]. The difference in RNA regulatory mechanisms between prokaryotes and eukaryotes may suggest that it is unlikely prokaryotic microbial exogenous RNA can be decoded using the eukaryotic silencing complex but other opportunities may exist where exogeneous regRNA acts not as a mediator of canonical gene silencing but rather binds to host receptors and activates signal transduction cascades [33]. Some symbiotic and pathogenic bacteria have also evolved the ability to colonize and live inside plant cells [35], therefore potentially creating a possibility for regRNA and effector proteins transfer between bacteria and cytoplasm of the plant cell. We propose that native exogenous regRNA exchange may exist between prokaryotic microorganisms and plant cells, although specific examples are not easily discoverable and future practical applications are yet to be invented.

Several reported examples mentioned above and reviewed elsewhere suggest a diversity of forms and mechanisms of exogenous regRNA transfer as outlined in Figure 1. Our current understanding of mechanisms of RNA secretion, transport and uptake by plant cells is still very limited. Different types of RNAs such as small regRNAs as well as long non-coding RNAs and messenger RNAs have been reported to move intercellularly and involved in cell-to-cell and long-distance signaling during plant development and response to environmental cues [36,37]. Plant plasmodesmata (PD) has been shown to play a critical role in intercellular trafficking of macromolecules, both RNA and proteins, across the cell wall [38]. It is still unclear if such intercellular trafficking is driven primarily by specific intrinsic properties of RNA or it is a non-selective diffusion-based process or combination of both. At least some mobile RNAs are associated with RNA binding proteins [36] that may facilitate selective secretion of certain RNAs through PD, although RNA binding proteins may also play a role in RNA stability. Interestingly, both endoplasmic reticulum and PD are involved in RNA virus movement in plants [39] suggesting that at least some eRNAi cases can be explained by RNA-protein complex trafficking similar to processes occurring during virus intercellular movement. 

In *C. elegans* and some insect species, long dsRNA uptake is driven by specific mechanisms such as SID2 and clathrin-mediated respectively [40,41]. While similar mechanisms have not yet been found to be involved in RNA uptake in plants, the clathrin-mediated endocytosis pathway has been shown to be responsible for uptake of exogenous dsRNA in the white mold phytopathogen *Sclerotinia sclerotiorum* [42]. Recent findings detailing new types of extracellular RNAs and mechanisms of RNA secretion in non-plant organisms [43,44] also suggest that we cannot exclude the possibility of discovering new mechanisms of naked regRNA or ribonucleoprotein complexes transfer, both by secretion and uptake, in plant cells in addition to the reported extracellular vesicles-mediated transfer of sRNAs.

Apart from downregulation of target genes, exogenous RNA may trigger receptor mediated regulatory signaling, as in case of dsRNAs inducing pattern-triggered immune signaling [45], which is an even less explored opportunity of the topical RNA strategy for practical applications. We also would like to note what while the systemic spread of the exogenous regRNA signal in organisms seems to be one of the most desirable characteristics for many practical eRNAi applications, the local regulation or signal initiation in specific cell types may have valuable practical applications in some cases as well. We have no doubt that leveraging natural mechanisms of RNA secretion, uptake and stabilization would allow for more efficient use of eRNAi for practical applications. The biological function of exogenous regRNA transfer is often difficult to elucidate due to technical challenges. We think it is likely that many new cases and forms of eRNAi will be identified and our understanding of the significance of this phenomenon on a global scale and direction of evolutionary processes is very far from complete. From an evolutionary point of view, eRNAi could be beneficial for plants during interactions with symbiotic microorganisms or as a plant defense against pathogenic microorganisms or herbivorous pests. At the same time, however, eRNAi may provide a way for pathogenic invaders to regulate gene expression in host cells. Interacting organisms can acquire the ability to uptake or deliver regRNA, but we cannot exclude the possibility that many cases of RNA transfer are an ancient relic process and may have a neutral effect. 

The more we understand the evolutionary trajectory of eRNAi cases the more we will understand the mechanisms and biological function of this process in different organisms. This knowledge of native mechanisms can be leveraged to develop practical applications, including crop protection from insect pests, microbial pathogens and weeds, plant adaptation to abiotic stresses, promoting plant-microbe symbiotic interactions, and other possibilities aimed at improving agriculture. 

Significant technical challenges remain to demonstrate unambiguously the functional role of exogenous RNA in heterologous organisms, especially when the recipient/host has limited exogenous RNA uptake capacity and lacks amplification and/or systemic spread of the RNA regulatory signal. In such cases, a phenotypic effect or impact on the host is difficult to measure due to the temporal and localized nature of the response and challenges in identifying target cells and gene products or affected pathways. Elucidating knowledge that regRNA transfer occurs but does not result in a biologically meaningful effects could still add value to the understanding of the evolution of regRNA, exogenous RNA mobility, uptake and eRNAi. Expanding our knowledge of regRNA biogenesis and function in donor and recipient organisms, increasing the amount of available genomic and transcriptomic data and onboarding advanced detection techniques would allow us to identify currently undetected cases of eRNAi by specific signature marks of exogenous regRNA processing or association with host RNA regulatory machinery. These approaches also require caution in the interpretation of results since because of the high sensitivity of next-generation sequencing technologies and the possibility of cross contamination. Careful evaluation of the most appropriate techniques or combination of methods and unbiased interpretation of results are critical steps to distinguish between false positives and true eRNAi as well as to minimize false negatives and missing true cases of eRNAi. Advanced sRNA sequencing approaches such as degradome sequencing, cross-linking immunoprecipitation and single cell RNA sequencing in combination with laser captured microdissection, microfluidic-based cell sorting, extracellular vesicle purification and new bioinformatic tools relying on extensive databases could help to identify new true cases of eRNAi and better understand the evolution of eRNAi in plants and interacting organisms. Significant progress has been made studying extracellular RNA as cell-to-cell and long distance signaling in various organisms [46,47] and some methods/approaches including computational ones should be adopted for studying eRNAi [48]. 

As the aforementioned techniques have become more broadly available and affordable in recent years, their application to eRNAi studies may help us to conduct large-scale surveys of eRNAi events not only in the laboratory but also under natural environmental conditions. One example of such a survey could be a sampling of herbivorous insects from a natural feeding environment and conducting sRNA sequencing analysis for the presence of exogenous plant-derived RNA within insects and confirmed to be processed by insect RNAi machinery into insect siRNAs. Mapping the data to appropriate plant and insect genomes and transcriptomes (Figure 2) similar to what was previously conducted on a smaller scale [19] would allow us to identify insect species and populations that are capable of exogenous RNA uptake under natural environmental conditions and potentially learn more about eRNAi evolution. A better comprehension of the eRNAi phenomenon on a global scale would not only help shed light on evolutionary aspects of this biological process but would also let nature guide us on better designs, broader ranges and more efficient uses and applications of eRNAi. 

## Figures and Tables

**Figure 1:**
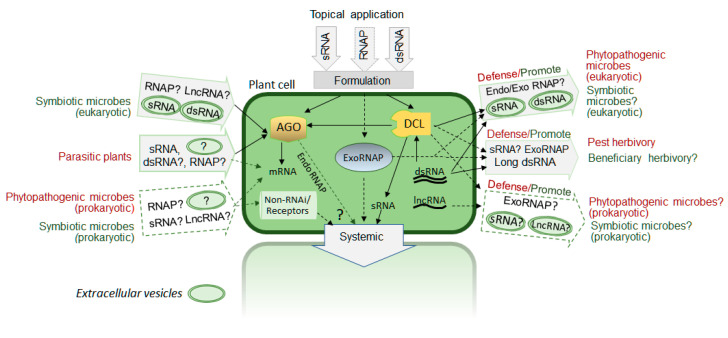
Multiple forms of exogenous RNA transfer between plant cell and biotic environment. Potential mechanisms, proposed biological roles and possible practical applications: red color font —pathogenic/defense interaction; green color font—symbiotic/beneficial interactions. Dashed line arrows and “?”—proposed (hypothetical) cases that are currently not fully supported by published reports; lncRNA—long non-coding RNA, endoRNAP—plant endogenous RNA/protein complex, exoRNAP—engineered RNA/protein complex, AGO—argonaute protein, DCL—dicer-like protein. dsRNA—double stranded RNA.

**Figure 2:**
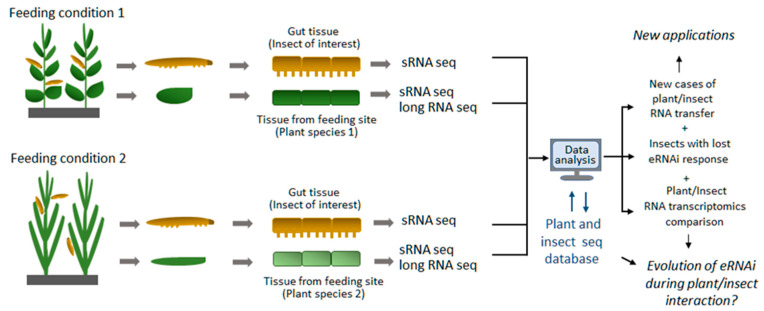
Proposed high throughput screening approach for identification of new cases of exogenous RNA transfer from plants to insects under natural insect feeding environment. Insect material to be collected from natural feeding sites along with correspondent plant material and subjected to Next-Gen Sequencing (sRNA and long RNA Seq). Data analysis to be used to identify plant-derived sRNAs in insect cells (especially gut cells) by comparison to plant sRNA and long RNA seq as well as insect endogenous sRNA dataset obtained from insects fed on different plant material (different plant species in upper and lower panels) or insect fed on artificial diet as a control.
